# Patients’ and professionals’ experiences with remote care during COVID-19: a qualitative study in general practices in low-income neighborhoods

**DOI:** 10.1017/S1463423624000240

**Published:** 2024-06-03

**Authors:** Jelena Kollmann, Shakib Sana, Tessa Magnée, Sarah Boer, Inge Merkelbach, Paul L. Kocken, Semiha Denktaș

**Affiliations:** 1 Erasmus School of Social and Behavioural Sciences, Erasmus University Rotterdam, Rotterdam, the Netherlands; 2 Municipality of Rotterdam, Rotterdam, the Netherlands

**Keywords:** accessibility, COVID-19, low-income neighborhoods, primary care, remote care

## Abstract

**Aim:**

To explore how patients and general practice professionals in low-income neighborhoods experienced the increase of remote care during COVID-19.

**Background:**

As the GP (general practitioner) is the first point of contact in Dutch health care, there are concerns about access to remote care for patients from low-income neighborhoods. Now that general practice professionals have returned to the pre-pandemic ways of healthcare delivery, this paper looks back at experiences with remote care during COVID-19. It investigates experiences of both patients and general practice professionals with the approachability and appropriateness of remote care and their satisfaction.

**Methods:**

In this qualitative study, 78 patients and 18 GPs, 7 nurse practitioners and 6 mental health professionals were interviewed. Interviews were held on the phone and face-to-face in the native language of the participants.

**Findings:**

Remote care, especially telephone consultation, was generally well-approachable for patients from low-income neighborhoods. Contrarily, video calling was rarely used. This was partly because patients did not know how to use it. The majority of patients thought remote care was possible for minor ailments but would also still like to see the doctor face-to-face regularly. Patients were generally satisfied with remote care at the time, but this did not necessarily reflect their willingness to continue using it in the future. Moreover, there was lack in consensus among general practice professionals on the appropriateness of remote care for certain physical and mental complaints. Nurse practitioners and mental health professionals had a negative attitude toward remote care. In conclusion, it is important to take the opinions and barriers of patients and care providers into account and to increase patient-centered care elements and care provider satisfaction in remote care. Integrating remote care is not only important in times of crisis but also for future care that is becoming increasingly digitalized.

## Introduction

During the first wave of the COVID-19 pandemic, physical access to general practice care was abruptly limited to contain the spread of the coronavirus (Verhoeven *et al.*, 2020; Matenge *et al.*, 2022). Most appointments were replaced by remote care, which was limitedly used prior to the pandemic. After the implementation of the COVID-19 measures in March 2020, Dutch general practices increased their use of various forms of remote care rapidly. For example, the use of e-consults was increased from 68% to 85% and the use of video calling from 0% to 72% (Keuper *et al.,* 2021). This was not only due to safety measures but also because patients were avoiding the care settings due to perceived risk of being infected with the coronavirus (Lazzerini *et al.,* 2020; Danhieux *et al.,* 2020).

Remote care constitutes all healthcare provision that substitutes direct face-to-face contact between the healthcare professional and the patient (Mann *et al.*, 2020). Telephone consultations, email, video calls, text messages, and eHealth applications can all be considered remote care (Sana *et al.,* 2022). The rapid implementation of remote general practice care on a large scale created a new situation for patients and for general practice professionals (these include general practitioners, or GPs, nurse practitioners, and mental health professionals).

This new situation led to concerns about the access to general practice care for patients from low-income neighborhoods for several reasons (Shadmi *et al.,* 2020). Firstly, these groups often experience financial difficulties and lower health literacy, which are associated with a higher need for a general practice appointment during the first wave of COVID-19 (Sana *et al.*, 2022). Secondly, studies show that low SES individuals tend to possess lower digital literacy, which deprives them from certain benefits that come with using digital health technologies (Guo, 2021; Western *et al.*, 2021). Thirdly, the prevalence of chronic diseases, such as diabetes mellitus and COPD (Jordan *et al.*, 2014; White *et al.,* 2016; Consolazio *et al.,* 2020) as well as psychosocial problems (Jordan *et al.*, 2014; Fone *et al.*, 2014), is higher in these groups. This ultimately leads to a higher number of general practice consultations, which is often their first point of trusted contact (Fone *et al.*, 2014; Sripa *et al.*, 2019; Murphy and Salisbury, 2020; Barlow *et al.,* 2021).

Accessibility to care can be defined as the opportunity to have healthcare needs fulfilled (Levesque *et al.*, 2013). In their paper, Levesque *et al.* (2013) mention several dimensions of accessibility. For the scope of this article, we will be focusing on the dimensions of approachability (ie, the ability to perceive) and appropriateness (ie, the ability to engage). To perceive remote care services, these need to be available and properly communicated to patients, and patients need a level of health literacy and knowledge about health and sickness. To engage with remote care services, care providers need to offer good quality services in a continuous way that are a proper fit with the person and their respective resources, values, and skills. From the patients’ side, this means participating in the decision-making about what treatment is a good fit and actively engaging in this treatment. This depends on patients’ empowerment, that is their level of self-efficacy, health literacy, and self-management, but also on their communication skills (Levesque *et al.*, 2013).

Safeguarding accessibility to care is an important part of patient-centered care (Institute of Medicine (US) Committee on Quality of Health Care in America, 2001; Levesque *et al.*, 2013; Rathert *et al.*, 2013). Patient-centered care encompasses providing care that is compassionate, empathetic, and responsive to the needs, values, and expressed preferences of each patient (Institute of Medicine (US) Committee on Quality of Health Care in America, 2001). This, in turn, has been shown to positively influence patient satisfaction, which is linked to higher well-being and higher adherence to treatments. Higher patient satisfaction has also been linked to a higher usage of remote care (Rathert *et al.*, 2013; Hawrysz *et al.*, 2021). However, healthcare systems have largely switched back to providing face-to-face care (Don, 2021). This might suggest that patients’ and general practice professionals’ satisfaction with remote care was not high.

This is problematic, because remote care could greatly save time and costs for both patients and professionals, when both are willing and capable of using it, enhance patient-centered care, empower patients, and benefit patients with chronic conditions (Beheshti *et al.*, 2022; DePuccio *et al.*, 2022; Marques *et al.*, 2022; Goldman *et al.*, 2023). For professionals, remote care could save time and costs by allowing the physician to work from home (DePuccio *et al.*, 2022). However, it is important to note that there is no clear evidence yet that remote care lowers the GPs’ workload (Van de Burg *et al.*, 2023). Remote care may also not save time and costs with all patients. However, for those cases in which the doctor can judge the complaint well remotely and the patient communicates well, telephone consultations could for instance cost less than face-to-face consultations. In addition, remote care might be able to enhance patient-centered care, because when patients are in favor of having contact with a general practice professional remotely and circumstances allow it, then this would meet their personal preference. However, it is important to note that it might not always be possible to adhere to the preferences of the patients, especially during challenging times such as a pandemic.

To ensure that remote care can be further developed and implemented in health care (Don, 2021), it is important to look at the experiences of patients and general practice professionals that have led them to largely return to the pre-pandemic ways of healthcare delivery. This is especially relevant for possible future crises and the further digitalization of health care. Moreover, to our knowledge, combining both patients’ and general practice professionals’ perspectives on remote care in a primary care setting during a pandemic has not yet been done, especially in low-income neighborhoods.

Therefore, our main objective is to explore how patients from low-income neighborhoods and general practice professionals experienced the sudden increase of remote care during the early stages of COVID-19. This will help us gain better understanding of opportunities for improvement of remote care in primary health care for the future. We will do this by looking at the approachability and use of remote care, identifying circumstances under which remote care was appropriate, and assessing how satisfied patients and professionals were.

## Methods

In 2020-2021, a qualitative study was conducted to investigate the experiences of patients and general practice professionals with different types of remote care during COVID-19 in low-income neighborhoods.

### Study design

#### Participant selection

This study was based on a phenomenological approach (Alhazmi and Kaufmann, 2022). Convenience sampling was used to recruit participants from the professional network of author SS. From this network, 15 general practices from Rotterdam and its surroundings participated in this study. We focused on including practices with patients from groups that face health disparities. These were located mainly in low-income neighborhoods, with a few practices from other neighborhoods for comparison. General practice professionals and patients were selected from these practices.

Professionals were approached face-to-face or by phone for participation in the study. Patients were approached face-to-face or by telephone by general practice professionals. We strived for a diversity in the patient population in ethnic background, level of health literacy, education, and chronic disease. A total of 109 participants, comprising of 78 patients and 31 professionals (18 GPs, 7 nurse practitioners, and 6 mental health professionals), participated in the study.

#### Setting

During the first wave of COVID-19, the general practitioners limited the physical access to the practices drastically, making remote care the main way of accessing general practice care. At the end of the first wave of COVID-19 (July 2020) in the Netherlands, the stringent measures were slowly liberalized. The physical access to the practice was partly restored, and the number of face-to-face consultations increased again.

#### Data collection

Data were collected over the phone during the first COVID-19 wave in the Netherlands, and shortly after (April–October 2020). No one else was present during the interviews besides the participant and researcher. Prior to being interviewed, all participants were informed about the aim of the study and methods of data collection and received information about the use and protection of their data. Patients gave verbal informed consent and professionals gave written informed consent.

The interview guide for the interviews with patients included the following themes: approachability to care (type of contact), appropriateness of care (opinion about contact, opinion about remote care, barriers to care), satisfaction with care, and background characteristics. The interview guide for interviews with professionals included similar themes as the patient interview guides: approachability to care (estimation of patient experience with contact), appropriateness of care (patient usage of care, barriers to care for patients), satisfaction (of professionals and estimated for patients, attitude toward remote care), and background information (Andersen and Newman, 2005; Babitsch *et al*., 2012; Levesque *et al.*, 2013). The themes overlapped in both interview guides for patients and professionals. Subsequently, interviews were conducted until saturation was achieved. Interviews lasted between 30 and 60 min and were audio-recorded and transcribed. Data were anonymized by assigning a number per patient and professional.

### Research team and reflexivity

#### Personal characteristics and relationship with participants

Qualitative interviews were conducted by PK, SB, IM (all senior researchers at the time of the study), two research assistants, and an external interview bureau. This bureau was hired to conduct interviews with patients in their native language. The interviewers had ample experience with conducting interviews. There were no prior relationships established between participants and interviewers, nor did participants know any characteristics of the interviewers. Authors JK and TM analyzed the data. Author SS is a medical doctor (MD) and general practitioner who was directly involved in all aspects of this study and whose experience was used throughout the project. Moreover, all authors were involved in research into behavior change and community care in collaboration with the municipality of Rotterdam (named Healthy’R).

### Analysis

#### Data analysis

The transcripts were coded in Atlas.ti version 8 and 9. The interviews were analyzed through directed content analysis. The patient interviews were coded by five coders (JK, IM, NS, FA, and SvP), and the general practice professionals’ interviews were coded by four coders (PK, SB, RS, and LM). Authors (IM, PK, and JK) provided the coders with a coding tree, as well as a description of the coding tree. The coding tree was developed, discussed, and adapted with the research team. The coding tree included deductive codes, and during the coding process, inductive codes were also added. Deductive coding was used to center the analysis around the theory. Inductive coding was used for deriving new relevant codes from the interviews and to categorize existing codes into broader codes or other relevant themes.

Firstly, deductive codes were used to code the interviews. For the patient interviews, the following codes were used: contact with general practice professionals, type of contact (specific codes per type, eg, video calling), opinion about contact (not satisfied, neutral, satisfied), barriers to contact with general practice professionals, opinion remote care (negative, mixed, positive), contact with the general practice, and background characteristics (eg, age). For the professionals’ interviews, the following codes were used: use of remote care, increased use of general practice, decreased use of general practice, groups for whom remote care was suitable, groups for whom remote care was less suitable, health complaints for which remote care was suitable, health complaints for which remote care was less suitable, alternative forms of care, self-limiting barriers, barriers to general practice care, accessibility, and background characteristics (eg, sex).

Secondly, some new codes were added inductively to the interviews (eg, no perceived barriers, other type of care, delayed care). Moreover, codes were simplified and overlapping codes and subcodes were combined into more general codes. The results of these codes were gathered in reports, which were summarized to gain the answers to the research questions (Skjott Linneberg and Korsgaard, 2019). In addition, four questions that were asked during the interviews with the general practice professionals about their views on remote care were summarized quantitatively with frequencies and percentages (see Table 1).

**Table 1.** GP professionals’ views on remote care (*n* = 31)

		All professionals (*n* = 31)		GPs (*n* = 18)		Nurse practitioners (*n* = 7)		Mental health professionals (*n* = 6)	
		*n*	(%)	*n*	(%)	*n*	(%)	*n*	(%)
Amount of provided remote care during COVID-19 crisis	< 80%	4	(16)	3	(20)	1	(25)	0	(0)
	>= 80%	21	(84)	12	(80)	3	(75)	6	(100)
Attitude toward remote care^a^	Positive	18	(58)	14	(78)	2	(29)	2	(33)
	Negative	13	(42)	4	(22)	5	(71)	4	(67)
Satisfaction with adjusted working methods	Very satisfied	3	(10)	1	(6)	0	(0)	2	(40)
	Satisfied	19	(66)	14	(82)	4	(57)	1	(20)
	Moderately satisfied	6	(21)	1	(6)	3	(43)	2	(40)
	Unsatisfied	1	(3)	1	(6)	0	(0)	0	(0)
	Very unsatisfied	0	(0)	0	(0)	0	(0)	0	(0)
Estimated patients’ satisfaction with adjusted working methods during the first COVID-19 wave	Very satisfied	1	(4)	0	(0)	1	(14)	0	(0)
	Satisfied	19	(70)	11	(73)	3	(43)	5	(100)
	Moderately satisfied	6	(22)	4	(27)	2	(29)	0	(0)
	Unsatisfied	1	(4)	0	(0)	1	(14)	0	(0)
	Very unsatisfied	0	(0)	0	(0)	0	(0)	0	(0)

a Based on three items on attitude toward remote care from the MIDI questionnaire (Fleuren *et al.*, 2014), that is pleasant, important, and suitable for my patients, with answer categories: highly agree (1), agree (2), neutral (3), disagree (4), highly disagree (5).

To avoid confirmation bias and enhance intercoder reliability, an intercoder agreement (ICA) test was performed to enhance intercoder reliability (in Atlas.ti referred to as intercoder agreement or ICA) (Hak, 2004; O’Connor and Joffe, 2020). For the patient interviews, this resulted in an ICA of 0.73. The professionals’ interviews had an ICA of 0.80. An ICA can lie between −1 and 1, so this study’s ICAs provide substantial intercoder agreement (Landis and Koch, 1977; O’Connor and Joffe, 2020).

## Results

### Characteristics of patients and general practice professionals

#### Patients’ characteristics

More than half of the patients were female (Table 2). The educational level of the patients was rated as low (ie, none, elementary or pre-vocational) for two-thirds of the sample. Two-thirds of participants had a non-native background and less than half had average to insufficient Dutch language proficiency. More than half of the patients reported having chronic health complaints and one-third of patients experienced financial difficulties to some extent. Two-thirds of the patients were unemployed.

**Table 2.** Characteristics of the interviewed patients (*n* = 78)

		*n*	(%)
Sex	Male	30	(39)
	Female	46	(61)
Education	None, elementary, pre-vocational	45	(60)
	Vocational	27	(36)
Dutch language proficiency	Good	48	(68)
	Medium	11	(16)
	Insufficient	11	(16)
Financial difficulty	Yes	21	(29)
	No	51	(71)
Chronic disease	Yes	42	(72)
	No	16	(28)
Contact with the GP	Face-to-face consultations	28	(29)
	Telephone consultations	28	(29)
	Calling for appointment	18	(20)
	Calling for medication	4	(4)
	Email	9	(9)
	Video calling	6	(6)
	Other care	2	(2)
	E-health	1	(1)

#### Professionals’ characteristics

Similarly, most interviewed professionals were female (Table 3). There were slightly more male professionals in the GP group. About half of GPs and two-thirds of mental health professionals had five to 10 years of working experience, whereas more than half of the interviewed nurse practitioners had less than five years of working experience. Also, the majority (80%–100%) of the professionals provided 80% or more remote care during the first months of COVID-19 (Table 1). Majority of GPs had a positive attitude toward remote care (78%), whereas considerably less nurse practitioners and mental health professionals had a positive attitude toward remote care (29% and 33% respectively). Most professionals were relatively satisfied with the adjusted working methods as caused by the COVID-19 restrictions at the beginning of the pandemic and estimated that patients would also be relatively satisfied with it (Table 1).

**Table 3.** Characteristics of professionals (*n* = 31)

		All professionals (*n* = 31)		GPs (*n* = 18)		Nurse practitioners (*n* = 7)		Mental health professionals (*n* = 6)	
		*n*	(%)	*n*	(%)	*n*	(%)	*n*	(%)
Sex									
	Male	9	(29)	8	(44)	0	(0)	1	(17)
	Female	22	(71)	10	(56)	7	(100)	5	(83)
Age									
	18–29	0	(0)	0	(0)	0	(0)	0	(0)
	30–39	12	(40)	8	(44)	4	(57)	0	(0)
	40–49	11	(37)	7	(39)	3	(43)	1	(20)
	50–59	3	(10)	0	(0)	0	(0)	3	(60)
	≥ 60	4	(13)	3	(17)	0	(0)	1	(20)
FTE									
	< 0.25	3	(10)	0	(0)	0	(0)	3	(50)
	0.25–0.5	3	(10)	1	(5)	1	(14)	1	(17)
	0.5–0.75	17	(55)	12	(67)	4	(57)	1	(17)
	≥ 0.75	8	(25)	5	(28)	2	(29)	1	(16)
Work experience (years)									
	< 5	8	(25)	2	(11)	4	(57)	2	(33)
	5–10	14	(45)	8	(44)	2	(29)	4	(67)
	10–15	4	(13)	3	(17)	1	(14)	0	(0)
	15–20	2	(6)	2	(11)	0	(0)	0	(0)
	20–25	0	(0)	0	(0)	0	(0)	0	(0)
	≥ 25	3	(11)	3	(17)	0	(0)	0	(0)

### Patients’ and professionals’ views on the approachability of the general practice

During the first weeks of COVID-19, the general practices were almost exclusively remotely approachable. According to Levesque *et al.*, (2013), approachability relates to people who face healthcare needs, and their ability to identify that certain healthcare services exist, can be reached, and have an impact on their health.

#### Patients’ views

The interviewed patients mentioned that they approached the general practice in different ways at the start of the COVID-19 pandemic. Many patients had contact with the general practice by telephone, either for a consultation, to make an appointment, or for a medication prescription (in order of most to least frequently mentioned) (see Table 2). One-third of the patients reported having a face-to-face consultation with the general practice professional, usually after a remote evaluation of the health complaint. Patient nr. 6: *‘And then we had to send a picture of my finger, based on that they would decide whether I should come by or not. I liked that, I thought that was a good idea’.* Only a small group of patients used video calling, and some patients said they searched for health information on the Internet. No patterns were found between patients’ background characteristics and their views on approachability.

#### Professionals’ views

Next to that, general practice professionals estimated the proportion of patients that approached the general practice and had contact remotely during the first weeks of the outbreak at a mean of 92%, which dropped to 65% during the following four months. Telephone consultation was the most frequent way that patients had contact with the general practice professionals, followed by email contact. GP nr. 15 about remote care: *‘Especially more emails with photos.…calling back and emailing back more often, choosing the moment at which we approach the patient’.*

### Appropriateness of remote care

According to Levesque *et al.*, (2013), appropriateness concerns the fit between remote care services and patients’ needs, its timeliness, the amount of care spent assessing the problems, and the quality of the service provided.

#### Patients’ views

Most patients were of the opinion that remote care could be useful and appropriate in some cases, when asked about the type of care that was used and their opinion on it, such as minor health complaints or complaints that do not require physical examination (Table 4). Patients mentioned that remote care was not appropriate for serious health complaints, such as cardiovascular problems. In some cases, regardless of the complaint, patients liked to be seen by the GP because they felt better if the doctor saw and treated them in person. Moreover, some patients believed that remote care was not appropriate for patients with insufficient Dutch language or for patients with low health literacy. Patient nr. 56: *‘Not everyone is able to contact their GP from a distance. There are people who cannot speak Dutch or are not skilled enough with health-related matters’.* In other cases, patients mentioned that remote care was not at all appropriate, as they needed face-to-face reassurance from their doctor. Patient nr. 1: *‘I believe the doctor can only help me when I visit face-to-face. Telephone consults are impersonal’.* Patients did not bring up the use of remote care for mental health issues. Moreover, no patterns were found in patients’ background characteristics and their views on appropriateness of remote care.

**Table 4.** Appropriateness of remote care for health complaints and patient subgroups according to professionals and patients

		According to professionals	According to patients
Health complaints	Appropriate	Complaints that do not require physical examination (such as stomach aches, or low back pain) Skin problems (patients can send pictures through email) Mental health problems (safe feeling, more openness amongst patients) Respiratory complaints (without alarm signals)	Minor health complaints that can be solved over the phone or with a photo through email (such as skin rashes)
	Less appropriate	Complaints that require physical examination, such as musculoskeletal system complaints, stomach aches, gynecological problems Mental health problems (less non-verbal communication through remote care) Skin problems (unclear pictures or video, still wanting to see or examine it live)	Complaints requiring physical examination (such as cardiovascular problems, certain wounds, and moles) Serious health complaints (such as cardiovascular diseases)
Patient subgroups	Appropriate care	Elderly (for check-up telephone consultations, when home visits were not feasible during the first wave, and for emailing with home care) Patients with a higher educational level Youth and middle-aged adult patients Patients with a job Patients with a (stable) chronic disease (for remote measurements) Patients who speak and understand the Dutch language well Patients who have digital skills	
	Less appropriate	Patients who do not speak and understand the Dutch language well Elderly (they prefer home visits, and GPs want to see them live) Patients with a lower educational level Patients without technological means Patients with a migration background	Patients who do not speak and understand the Dutch language well

In relation to the utilization of remote care, patients mentioned a lack of (communication) technology or digital skills to use them, diminishing their ability to choose the proper service type for their health needs and actively engaging in it. Patients also mentioned that they often waited before contacting the GP. In the meantime, they waited for the health complaint to pass or they tried to solve it in another way, for example by themselves, by seeking alternative types of care, or by asking for advice from their family or friends. Patient nr. 47: *‘There are things that pass on their own, such as a sore throat or pain in the ear. Then I think: oh right, I know these symptoms’.*

#### Professionals’ views

Comparably, general practice professionals mentioned that remote care is not appropriate for complaints that require physical examination. Their opinions were mixed on which health complaints required physical examination. For example, there was no consensus in the interviews about whether stomachaches, musculoskeletal problems, and low back pain required physical examination.

Moreover, professionals discussed the appropriateness of remote care for mental health consults. Opinions were also mixed on this matter. Some found mental health problems to be suitable for remote care, mainly because patients felt safe to open up. However, according to other professionals, remote care was not suitable for mental health consultations because of a lack of non-verbal communication. GP nr. 7: *‘I think that my intuition is functioning less well through remote care. On the other side, I suppose some people may talk easier from behind a screen’.*

Next to the type of health complaint, the appropriateness of remote care was also determined by certain patient characteristics according to general practice professionals. Remote care was appropriate for highly educated patients and less suitable for (some) elderly, and for patients with a migration background, insufficient Dutch language proficiency, a low level of education, and/or without digital skills.

According to general practice professionals, some patients struggled to use remote care due to a lack of appropriate technology. GP nr. 6: *‘I have patients who do not have an email address or a smartphone. They do exist. You should offer a broad range of possibilities in the type of neighborhood I work in’.* Moreover, many patients did not know how to video call, or they did not like it. In other cases, GPs thought video calling did not add anything extra. Due to this, the option for video calling was often not offered.

### Satisfaction with remote care

#### Patients’ views

The majority of patients were satisfied with the contact they had with the general practice professional during the first wave of COVID-19. Patients even experienced benefits from remote care. Patient nr. 37: *‘It was nice because it is done quickly, and you do not have to go anywhere for it’.* However, a few patients who received remote care said they were aware that it was necessary at the moment, but they actually preferred face-to-face contact. Patient nr. 11: *‘It is what it is right now, but I would rather come by for a face-to-face consultation’.* Some patients thought remote care was rather impersonal. They experienced less attention for their health complaint and less engagement from the general practice professional. Also, practical issues arose, such as difficulty explaining their health complaint over the telephone due to language barriers, time-consuming explanation of complaints through email or telephone, long waiting times on the telephone, and costs of telephone use. Patient nr. 13: *‘The assistant put me on hold for half an hour, and when it was finally my turn, she told me the doctor is busy again. I really did not like that’.* Nevertheless, the majority believed remote care was useful for small complaints or for a quick question, but they would also still want to be seen by the doctor occasionally (especially with serious concerns). Patient nr. 57: ‘*Sometimes you have to see your GP in person…you can’t see how someone is doing physically or mentally over the phone’.*

#### Professionals’ views

Looking at the general practice professionals, their satisfaction with remote care varied (Table 1). Nurse practitioners and mental health professionals had a generally negative attitude toward remote care. They were also the least satisfied with the adjusted working methods during the first wave of COVID-19 and often estimated that patients would be moderately satisfied (Table 1). Some mental health professionals shared that they had a hard time understanding their patient due to a bad connection or due to a language barrier. They also could not see their patient’s body language, which added to their concerns about the quality of care. Mental health professional nr. 83: *‘Sometimes the connection is not good, then you must ask, “Could you repeat that?” … and sometimes you think “I actually did not hear half of it clearly.” That is a big disadvantage’.* Mental health professionals also mentioned that telephone consultations were more tiring for them than face-to-face consultations.

On the other hand, GPs had a more positive attitude toward remote care. They were satisfied with the adjusted working methods or thought their patients would be satisfied, given the circumstance of the COVID-19 pandemic.

## Discussion

The objective of this study was to investigate the experiences regarding approachability, appropriateness, and satisfaction of both patients and general practice professionals in low-income neighborhoods with remote primary care during the first wave of COVID-19.

Within a short time, the use of remote care has increasingly been implemented in primary care instead of face-to-face care. Most patients seemed to be able to make use of remote care; however for a few, this proved challenging. Patients with low Dutch language proficiency and low health literacy and digital literacy struggled with using remote care (especially video calling). One patient also did not have the means to use remote care. Remote care is also not appropriate for certain health complaints that require physical examination. The majority of patients thought remote care was possible for minor ailments but would also still like to see the doctor face-to-face regularly. Patients were generally satisfied with remote care at the time, but this did not necessarily reflect their willingness to continue using it in the future. Moreover, general practice professionals mentioned that some patients did not like video calling, and most GPs believed that it had little additional value to ordinary telephone calls. Mental health professionals found it difficult to understand patients when using remote care, and they had a negative attitude toward remote care. It is important to keep in mind that this was the view during the first phase of a sudden implementation of remote care and that with time and the right equipment, guidelines, skills, and willingness, remote care could be more deployable in general practices in low-income neighborhoods.

Despite finding that remote care was generally well-approachable, it seemed to discourage some patients to seek help until their symptoms progressed. These patients managed their health problems on their own during COVID-19. However, it is important to note that we cannot rule out the possibility that waiting for symptoms to get worse before seeking help might be a patient’s natural inclination and therefore unrelated to the limitations in care due to the COVID-19 pandemic.

Video calling was not widely used by patients in this study. This was partly because some patients were not willing to do so and some expressed resistance to it. One study (Mueller *et al.,* 2020) investigated the difference between patients with and without experience with video calling. Patients with experience had a more positive view of its benefits and use, while those without valued it less. This might offer an explanation for why some patients in this study were unwilling to use video calling, as they were new to it and did not have any prior experience with it. Another study shows that before the pandemic, patients preferred video consultations less than 50% of the time (Gilbert *et al.,* 2020). This shows that remote care was acceptable in a crisis, but that most patients preferred a face-to-face consult for their next appointment. Part of the reason for this preference, according to both Gilbert *et al.* (2020) and our study, is the fact that patients believed remote care was impersonal. This illustrates the need for more personalized patient-centered care in remote care, including key elements of patient-centered care, such as respecting patients’ preferences, ensuring access to care, and communicating well (Rathert *et al.*, 2013). These elements might be introduced in remote care by asking patients about their preferred type of remote care, providing access to remote care, and practicing patient-centered communication. This type of communication includes asking about the patient’s needs, values, and perspective, giving the patient the proper information to participate in their care, and building trust and understanding between patient and care provider (Levinson *et al.*, 2010).

General practice professionals were also less willing to use video calling. Hvidt *et al.* (2023) reported similar findings in their study. They suggest that this might be due to perceived barriers in general practices, such as communicative challenges, poor user-friendliness, and lacking technology and financial support for general practitioners to adequately carry out video consultations. This might also explain why general practice professionals in our study did not use video calling often.

GPs had mixed views on whether remote care was appropriate for certain physical complaints (such as stomachaches, musculoskeletal problems, and low back pain) and mental health problems. Literature suggests that for rheumatic and musculoskeletal diseases, telehealth can be used in screening as part of determining whether the patient needs to be referred (de Thurah *et al.,* 2022). It can also be used for monitoring disease, regulation of medication dosages, and in certain interventions that lack the use of medication. Patients with such diseases should be offered training in remote care for the proper use of its benefits (de Thurah *et al.,* 2022). Moreover, remote care can be used to self-manage chronic lower back pain (Yang *et al.,* 2019). It can also be used in addition to physiotherapy (Yang *et al.,* 2019). These studies show that remote care can be used in several ways when it comes to certain physical ailments.

Witteveen *et al.* (2022) showed that one of the main barriers for the use of remote care for mental health problems was poor technological literacy and the beliefs about reduced therapeutic alliance, particularly in the case of severe mental health disorders. Therapeutic alliance is an agreement on the goals and tasks of the therapy, and it can function to increase the bond between care provider and patient (Simpson and Reid, 2014). In this study, general practice professionals found it harder to notice more subtle signals of non-verbal communication from their patients, especially for mental health problems. Moreover, mental health professionals found it difficult to understand their patients verbally through remote care. This illustrates the need to improve remote care, for example through delivery, to be able to improve the verbal and non-verbal communication and increase therapeutic alliance between care provider and patient.

Patient satisfaction has been linked with an increased adoption of remote care (Kissi *et al.,* 2020). Ramaswamy *et al.* (2020) showed that patient’s satisfaction with video calling consults was high. It did not form a barrier for the use of remote care. This contrasts with our study, as patients were generally unable or unwilling to use video calling and wished to return to face-to-face care. However, Ramaswamy *et al.* (2020) did not take socio-economic background into account, which makes it hard to generalize and compare this finding. Our study found different results, but because we include low socio-economic background as opposed to some literature, we cannot properly compare our findings yet.

### Strengths and limitations

One of the significant strengths of our study is that we managed to conduct many interviews with patients from low-income neighborhoods, which are generally hard to reach. Moreover, we managed to include the perspectives of busy general practice professionals in this study, even during the first wave of COVID-19, which was one of the most challenging times in health care.

One of the limitations of our study is that the patients were mainly recruited via convenience sampling. This might have led to less representation of certain groups in our study population and selection bias. However, we managed to get an adequate range of different groups of patients with chronic health complaints, from migrant backgrounds and with limited health literacy, living in low-income neighborhoods. Respondents of these groups gave ample insight into all aspects of remote care utilization during a crisis for patients with a disadvantaged background. Moreover, results are representative for times when severe health crises take place. Next to that, there might have been a recall bias for the interviewed patients. However, the interview period was within maximum of 6 months after the start of the pandemic and questions were asked about the experiences during this COVID-19 period.

### Implications for practice

Remote care was generally approachable for most patients. However, some patients experienced problems with accessing it. To facilitate and enhance access to care for these patients, it is important for general practice professionals and government policymakers to consider barriers such as insufficient language proficiency, digital skills, digital means, and the willingness to use remote care (Houlding *et al.*, 2021; van Grootven *et al.*, 2022).

It is still unclear in what cases remote care can be most appropriate. At the time of the COVID-19 crisis, there was a lack of consensus as to which health complaints can be appropriately dealt with by providing remote care. The crisis makes it evident that clearer professional guidelines and policy are required to inform general practice professionals about the use of remote care, so they can reach a consensus for which physical and mental health ailments they can implement remote care (Kursīte *et al.*, 2022).

The attitude of nurse practitioners and mental health professionals toward remote care was predominantly negative. They feared that switching to telephone consultations would reduce the quality of care. This is partly the reason why they preferred to return to face-to-face care after the pandemic. Currently, healthcare systems have indeed mostly returned to face-to-face care. To integrate remote care into healthcare systems, it is important to take the opinions and barriers of patients and general practice professionals into account to secure patient-centered access to remote care and empower patients and professionals to use remote care when possible and useful (Kissi *et al.*, 2020; Althumairi *et al.*, 2022).

We also found that patient satisfaction with receiving remote care did not necessarily indicate a willingness to use it in the future. To increase acceptance, it is important to make remote care more personalized and targeted for patients who are willing and able to use remote care (Gilbert *et al.*, 2020; Record *et al.*, 2021). For these patients, a hybrid approach, combining both remote care and face-to-face care, could prove helpful in achieving this.

### Implications for research

For future research, it is important to study on which occasions remote care could be most appropriate, so proper guidelines can be made for the use of remote care (Kursīte *et al.*, 2022). Moreover, many patients prefer face-to-face care out of personal beliefs. More insight into why patients have these beliefs when it comes to the type of care they receive is needed. This might provide further insight for empowering patients from low-income neighborhoods to partake more in remote care. In addition, there is a discrepancy between GPs’ and patients’ views on patients’ usability of video calling for a health complaint. More research is needed to understand this discrepancy.

### Conclusion

Remote care, especially telephone consultations, during the first wave of COVID-19 was generally found to be approachable by patients from low-income neighborhoods. However, it was not appropriate for all patients. For instance, patients with low Dutch language proficiency or low digital literacy could not use remote care properly. The majority of patients thought remote care was possible for minor ailments but would still like to see the doctor face-to-face regularly. Patients were generally satisfied with remote care at the time, but this did not necessarily reflect their willingness to continue using it in the future. General practice professionals were also generally satisfied but said to enjoy face-to-face care more. Additionally, mental health professionals had a negative attitude toward remote care and found it difficult to understand patients clearly when using it. To stimulate patients and general practice professionals to use remote care when possible and useful, it is important to take the opinions and barriers of both parties into account and increase patient-centered care elements and care provider satisfaction in the use of remote care. Moreover, to build consensus among general practice professionals, creating guidelines for remote care is recommended. Integrating remote care with primary care is not only important in times of crisis but also for future care that is becoming increasingly digitalized.
